# Biofunctional soyasaponin Bb in peanut (*Arachis hypogaea L*.) sprouts enhances bone morphogenetic protein‐2‐dependent osteogenic differentiation via activation of runt‐related transcription factor 2 in C2C12 cells

**DOI:** 10.1002/ptr.6341

**Published:** 2019-03-18

**Authors:** Shin‐Hye Kim, Heung Joo Yuk, Hyung Won Ryu, Sei‐Ryang Oh, Duk Young Song, Kwang‐Sik Lee, Kie‐In Park, Sik‐Won Choi, Woo Duck Seo

**Affiliations:** ^1^ Forest Biomaterials Research Center National Institute of Forest Science (NIFS) Jinju Korea; ^2^ Department of Biological Sciences, College of Natural Science Chonbuk National University Jeonju Korea; ^3^ Korean Medicine Convergence Research Division Korea Institute of Oriental Medicine (KIOM) Daejeon Korea; ^4^ Natural Medicine Research Center Korea Research Institute of Bioscience & Biotechnology Cheongju Korea; ^5^ Division of Crop Foundation National Institute of Crop Science (NICS), Rural Development Administration (RDA) Wanju Korea; ^6^ College of Crop Science and Biotechnology Dankook University Cheonan Korea

**Keywords:** BMP‐2, bone, osteoblasts, peanut sprouts, Runx2, soyasaponin Bb

## Abstract

Improvement of bone formation is necessary for successful treatment of the bone defects associated with osteoporosis. In this study, we sought to elucidate the osteogenic activity of peanut sprouts and their bioactive components. We found that peanut sprout water extract (PSWE) enhanced bone morphogenetic protein‐2‐mediated osteoblast differentiation in a dose‐dependent manner by stimulating expression of runt‐related transcription factor 2 (Runx2) via activation of AKT/MAP kinases. We identified a major component of PSWE, soyasaponin Bb, as the bioactive compound responsible for improvement of anabolic activity. Soyasaponin Bb from PSWE enhanced expression of the osteogenic transcription factor Runx2 and alkaline phosphatase. The soyasaponin Bb content depended on sprouting time of peanut, and the anabolic action of PSWE was dependent on soyasaponin Bb content. Thus, PSWE and soyasaponin Bb have the potential to protect against bone disorders, including osteoporosis.

## 1 INTRODUCTION

Osteoporosis can increase the risk of bone fracture and is most frequently observed in elderly and post‐menopausal women. Bone fracture is associated with economic burden, pain, and skeletal deformity and is a serious and growing public health issue (Jachna, Shireman, Whittle, Ellerbeck, & Rigler, 2005).

Osteoporosis is caused by an imbalance between the activities of bone‐forming osteoblasts and bone‐resorbing osteoclasts (Takayanagi, 2005). Accordingly, control of bone formation and/or bone resorption represents a promising strategy for treatment of bone metabolic disorders such as osteoporosis. In clinical practice, treatment efforts have focused on anti‐resorptive agents, but these have not been sufficiently effective. Accordingly, new strategies for inducing osteoblast differentiation are required.

Osteoblast differentiation is a pivotal event in bone formation. Differentiation of osteoblasts from mesenchymal progenitor cells contributes to bone formation by promoting the production of extracellular matrix, which supports ossification by closely packed sheets on the bone surface (Beloti & Rosa, 2005). Osteoblast differentiation is regulated by signaling cascades and several transcriptional factors that promote mineralization and formation of bone. Runt‐related transcription factor 2 (Runx2), a transcription factor, is essential for osteoblast differentiation via its ability to induce the expression of osteoblastic downstream effectors (Komori, 2011). Furthermore, Runx2 is a pivotal mediator of signaling molecules including bone morphogenetic proteins, multifunctional growth factors belonging to the transforming growth factor beta superfamily (Chen, Zhao, & Mundy, 2004; Wang et al., 2006). Accordingly, activation of Runx2 represents a therapeutic strategy for treating osteoporosis with bone defect.

Plant‐derived natural products are widely used as complementary and alternative therapies for many diseases, including osteoporosis (Jiang et al., 2015). A simple and effective tool for improving biological activity is sprouting, which increases the levels of bioactive and nutritional components in seeds. For example, sprouting of peanut increases the abundance of bioactive components including resveratrol, isoflavones, and polyphenols (Kim, Park, & Lim, 2011; Wang et al., 2005). Furthermore, peanut sprouts have neuroprotective, anti‐oxidative, and anti‐obesity activities (Kang et al., 2010; Kang, Ha, Woo, & Kim, 2014; Lertkaeo et al., 2017). However, previous studies have not explored the effects of peanut sprout extract (PSE) on osteogenic differentiation or the molecular mechanisms underlying any such effects.

In this study, we investigated the anabolic activity of PSE and its pharmaceutical components on bone morphogenetic protein‐2 (BMP‐2)‐mediated osteoblast differentiation and optimized the osteogenic effect of peanut sprout water extract (PSWE) by manipulating sprouting time. Investigation of the mode of action revealed that PSWE and soyasaponin Bb, a bioactive component of the extract, potentiated the osteogenic mechanism.

## 2 MATERIALS AND METHODS

### 2.1 Preparation of peanut sprout extract

Sinpalkwang peanut (Arachis hypogaea L.) seeds were cultivated in 2016 in the experimental field at the National Institute of Crop Science, Jeonbuk, Korea. Peanut seeds were washed, incubated in water at 20°C for 18 hr, and then germinated at 65% humidity at 25°C in the dark. After harvesting 13 days after germination, peanut sprouts were immediately washed with clean sterile water and then freeze dried at −70°C. The dried sprouts (1.0 kg) were extracted with water, prethanol, or hexane (three extractions, 10 L each) in a shaking incubator for 2 days at 40°C. The extracts were filtered and evaporated under a vacuum and subsequently freeze dried to yield 112 g of water extract (11.2%), 85 g of prethanol extract (8.5%), and 45 g of hexane extract (4.5%) as dried powder. The concentrated extract was suspended in water, prethanol, or hexane to a final concentration of 100 mg/ml. The stock solution was further diluted in phosphate‐buffered saline (PBS).

### 2.2 Reagents and antibodies

Recombinant human bone morphogenetic protein‐2 (rhBMP‐2) was purchased from R&D Systems (Minneapolis, MN, USA). Penicillin, streptomycin, cell culture medium, and fetal bovine serum (FBS) were purchased from Invitrogen Life Technologies (Carlsbad, CA, USA). Antibodies against the following proteins were purchased from the indicated companies: actin, Smad, and secondary antibodies (Santa Cruz Biotechnology, Dallas, TX, USA); p‐Smad, p‐AKT, AKT, MAP kinases, and glyceraldehyde 3‐phosphate dehydrogenase (GAPDH; Cell Signaling Technology, Beverly, MA, USA).

### 2.3 Cell culture

All experiments were performed as described previously (Choi et al., 2017) with some modifications. Mouse mesenchymal precursor C2C12 cells were obtained from the American Type Collection (Manassas, VA, USA). C2C12 cells were maintained in alpha minimum essential medium (α‐MEM) containing 10% FBS, 100 U/ml penicillin, and 100 μg/ml streptomycin. To differentiate C2C12 into osteoblasts, the cells were seeded and allowed to attach and grow for 1 day, after which the medium was replaced with differentiation medium (α‐MEM containing 5% FBS and 100 ng/ml rhBMP‐2). The medium was changed every 3 days. Osteoblastic bone formation was monitored by alkaline phosphatase (ALP) staining.

### 2.4 ALP staining and activity assays

ALP activity of C2C12 cells was assessed using the ALP staining and ALP activity detection kit (Sigma‐Aldrich, St. Louis, MO, USA). Briefly, C2C12 cells were cultured under osteogenic differentiation conditions in the presence of vehicle, PSE, or soyasaponin Bb. After differentiation for 3 days, cells were washed twice with PBS, fixed with 10% formalin in PBS for 5 min, rinsed with deionized water, and stained with the ALP staining kit or measured using the one‐step p‐Nitrophenyl Phosphate (PNPP) substrate solution (Thermo Scientific, Waltham, MA, USA).

### 2.5 Cell proliferation assay

C2C12 cells were plated on 96‐well plates in triplicate. After treatment with PSE or soyasaponin Bb, the cells were incubated for 3 days and then cell viability was measured using the Cell Counting Kit 8 (CCK‐8; Dojindo Molecular Technologies, Rockville, MD, USA).

### 2.6 RNA isolation and real‐time polymerase chain reaction analysis

Primers were chosen using the Primer3 online tool. Primer sets used in this study are shown in Table S1. Total RNA was extracted from C2C12 cells using Trizol reagent (Invitrogen). First stand cDNA was synthesized using the RevertAid First Strand cDNA Synthesis Kit (Thermo Scientific). Real‐time PCR was performed using Applied Biosystems Power‐Up SYBR green PCR master mix (Thermo Scientific) and detected using Quantstudio®5 Real‐Time PCR (Thermo Scientific). The gene encoding *GAPDH* was used as an internal standard. All reactions were performed in triplicate, and data were analyzed using the 2^−ΔΔCt^ method (Livak & Schmittgen, 2001).

### 2.7 Western blotting

C2C12 cells were washed with ice‐cold PBS and lysed in lysis buffer (Cell Signaling Technology) supplemented with protease inhibitors (Roche, Basel, Switzerland). After centrifugation at 15,000 × *g* for 15 min, the protein in the supernatant was quantified using the detergent compatible protein assay kit (Bio‐Rad, Hercules, CA, USA). The quantified proteins were denatured, separated by sodium dodecyl sulfate‐polyacrylamide gel electrophoresis on 4–12% gradient gels, and transferred onto a polyvinylidene difluoride membrane using the iBlot 2 Dry Blotting System (Thermo Scientific). Blots were incubated with primary antibodies in 1% BSA overnight at 4°C and then incubated with secondary antibodies in 5% skim milk at room temperature for 2 hr. The membranes were developed using SuperSignal West Femto Maximum Sensitivity Substrate (Thermo Scientific) and visualized on a LAS‐4000 luminescent image analyzer (GE Healthcare Life Sciences, Little Chalfont, UK). Actin and GAPDH were used as loading controls.

### 2.8 Ultra‐high performance liquid chromatography‐charged aerosol detection (UHPLC‐CAD) analysis and isolation of soyasaponin Bb from PSWE

For the analysis of the main components, PSWE was dissolved at 1 mg/ml in methanol, filtered through 0.2‐μm filter units, and then subjected to UHPLC‐CAD analysis. Soyasaponin Bb analysis was conducted using a reverse‐phase UHPLC (Dionex Ultimate 3000, Thermo Scientific) equipped with an Acclaim™ RSLC Polar Advantage II (2.2 μm, 120 Å, 2.1 × 150 mm) column. The mobile phase was 0.1% acetic acid in water (A) or 0.1% acetic acid in acetonitrile (B). Solvent flow rate was 0.7 ml/min, and the column temperature was set to 35°C. The gradient was as follows: 0–2 min, 10% B; 5 min, 20% B; 15 min, 30% B; 20 min, 30% B; 40 min, 70% B; 50 min, 100% B; 50.1 min, 10% B; held for 9.9 min before returning to the initial conditions. Following injection of 2 μl of sample, eluted soyasaponin Bb was detected using UHPLC‐CAD (Corona Veo, Thermo Scientific). The soyasaponin Bb standard was purchased from ChemFaces (Wuhan, China). For isolation, the combined PSWE was evaporated under vacuum to yield a dark green gum (120 g). The resulting residue was subjected to chromatography on C_18_ silica gel (12 × 50 cm, 230–400 mesh, 120 g) using a series of water‐methanol mixtures [3:1 (*v*/v), 2 L; 2:1, 1.5 L; 1:1, 1.5 L; 1:1.5, 1.5 L] to yield five fractions (F1–F5). Fraction F3 (5.2 g) was fractionated by C_18_ silica gel column chromatography (3.0 × 40 cm, 230–400 mesh, 60 g) with a water‐acetonitrile mixture to yield 10 subfractions. On the basis of a comparison of TLC patterns, Subfractions 5–7 were evaporated to yield soyasaponin Bb (65 mg). For identification, evaporated subfractions were dissolved in methanol to 1 mg/ml, filtered through 0.2 μm filter units, and then subjected to UHPLC‐CAD analysis.

### 2.9 UPLC‐Q‐TOF/MS analysis

The identification of component peaks was carried out on an ultra‐performance liquid chromatography (UPLC) system (Waters, Milford, MA, USA) equipped with a photo‐diode array detector. Aliquots (2.0 μl) of test sample were then injected into an analytical column (BEH C_18_, 2.1 × 100 mm, 1.7 μm, Waters) at a flow rate of 0.4 ml/min. The mobile phase consisted of water containing 0.1% formic acid (A) and acetonitrile containing 0.1% formic acid (B). The linear gradient was as follows: 0–1 min, 10% B; 1–5 min, 10–40% B; 5–9 min, 40–60% B; 9–11.2 min, 60–100% B; 11.2–13.2 min, 100% B; 13.2–15 min; return to 10% B. The quadrupole time‐of‐flight mass spectrometer (Q‐ToF Premier™, Waters) was operated in both positive‐ and negative‐ion mode under the following conditions: capillary voltage, 2.5 kV; cone voltage, 40 V; source temperature, 110°C; desolvation temperature, 350°C. A sprayer with a reference solution of leucine‐enkephalin ([M + H]^+^ *m/z* 556.2771 and [M–H]^−^ *m/z* 554.2615) was used as the lock mass.

### 2.10 Statistical analysis

All quantitative values are presented as means ± standard deviation. Each experiment was performed in triplicate three to five times. Several figures show results from one representative experiment. Statistical differences were analyzed using Student's *t* test, and a value of *p* < 0.05 was considered significant.

## 3 RESULTS

### 3.1 PSWE stimulates BMP‐2‐induced osteoblast differentiation in C2C12 cells

**Figure 1:**
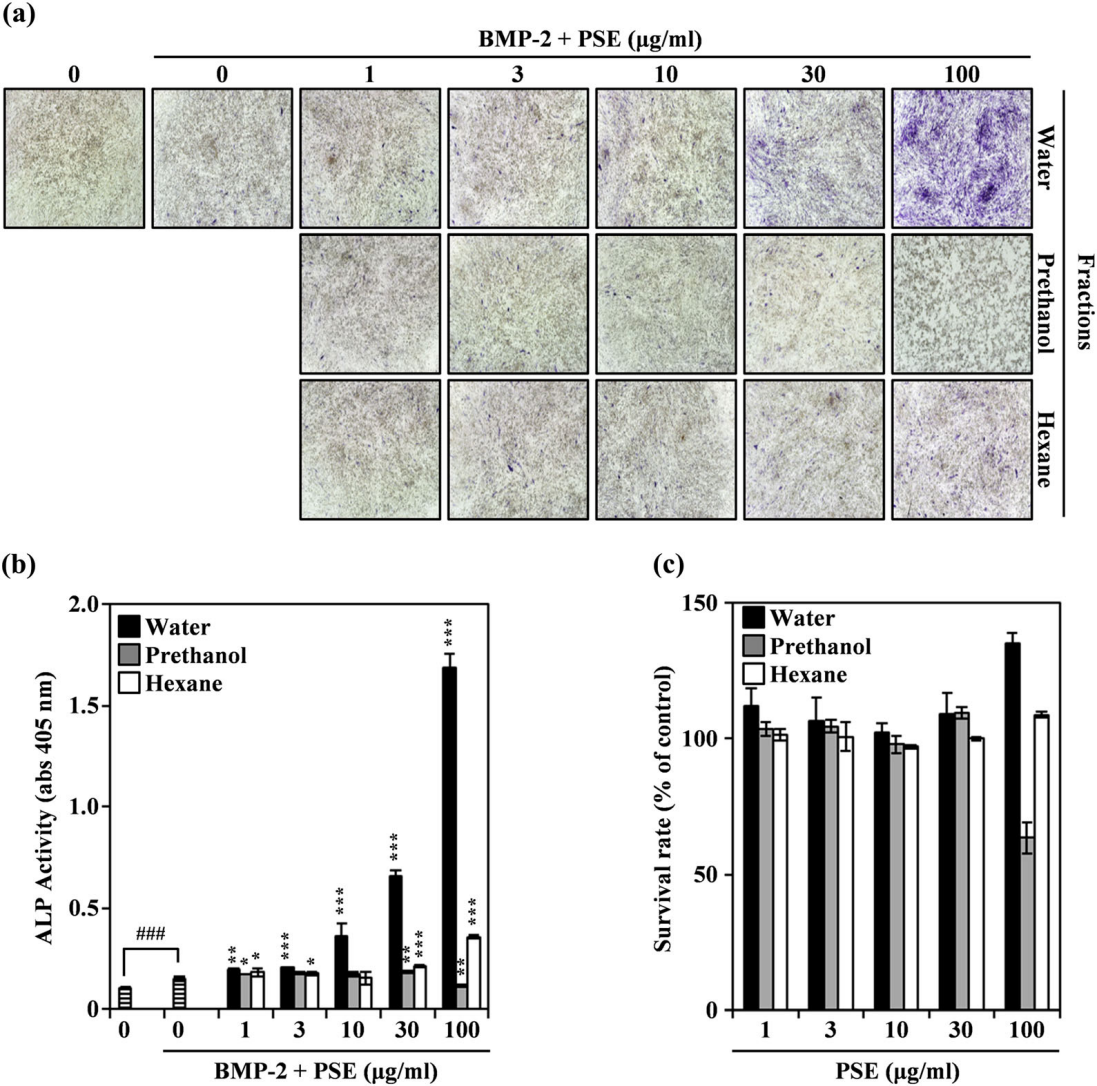
Peanut sprout water extract (PSWE) promotes bone morphogenetic protein‐2 (BMP‐2)‐mediated osteogenesis. (a) C2C12 cells were cultured for 3 days in the presence of BMP‐2 (100 ng/ml) with the indicated concentration of PSWE, peanut sprout prethanol extract (PSPE), or peanut sprout hexane extract (PSHE). Osteoblast differentiation was visualized by alkaline phosphatase (ALP) staining. (b) ALP activity was monitored by measuring absorbance at 405 nm. ^###^ *p* < 0.001 (versus control); *** *p* < 0.001 (versus BMP‐2‐treated group). (c) Effects of PSWE, PSPE, and PSHE on the viability of C2C12 cells were evaluated using the CCK‐8 assay. Data are expressed as means ± *SD* and are representative of at least three experiments [Colour figure can be viewed at wileyonlinelibrary.com]

To study the effect of PSE on BMP‐2‐mediated osteogenesis, we incubated C2C12 cells with varying concentrations of PSE fractions and then treated them with 100 ng/ml BMP‐2. As shown Figure 1a and Figure S1, PSWE dramatically induced the expression of ALP in a dose‐dependent manner in the presence of BMP‐2, whereas the prethanol and hexane fractions of PSE possessed only weak induction activity. Furthermore, PSWE enhanced bone mineralization in MC3T3‐E1 and C2C12 cells (Figure S2). However, ALP expression and bone mineralization in MC3T3‐E1 cells were not stimulated by PSWE in the absence of BMP‐2 (Figure S3). Consistent with this result, PSWE significantly increased BMP‐2‐stimulated ALP activity in a dose‐dependent manner (Figure 1b). At concentrations up to 100 μg/ml, PSWE and peanut sprout hexane extract (PSHE) did not exhibit any cytotoxicity, whereas peanut sprout prethanol extract (PSPE) inhibited C2C12 cell proliferation at 100 μg/ml (Figure 1c). Together, these results indicate that PSWE enhances BMP‐2‐dependent osteogenic differentiation to a greater extent than PSPE or PSHE.

### 3.2 PSWE promotes BMP‐2‐stimulated expression of transcription factors such as Runx2 during osteoblast differentiation

**Figure 2:**
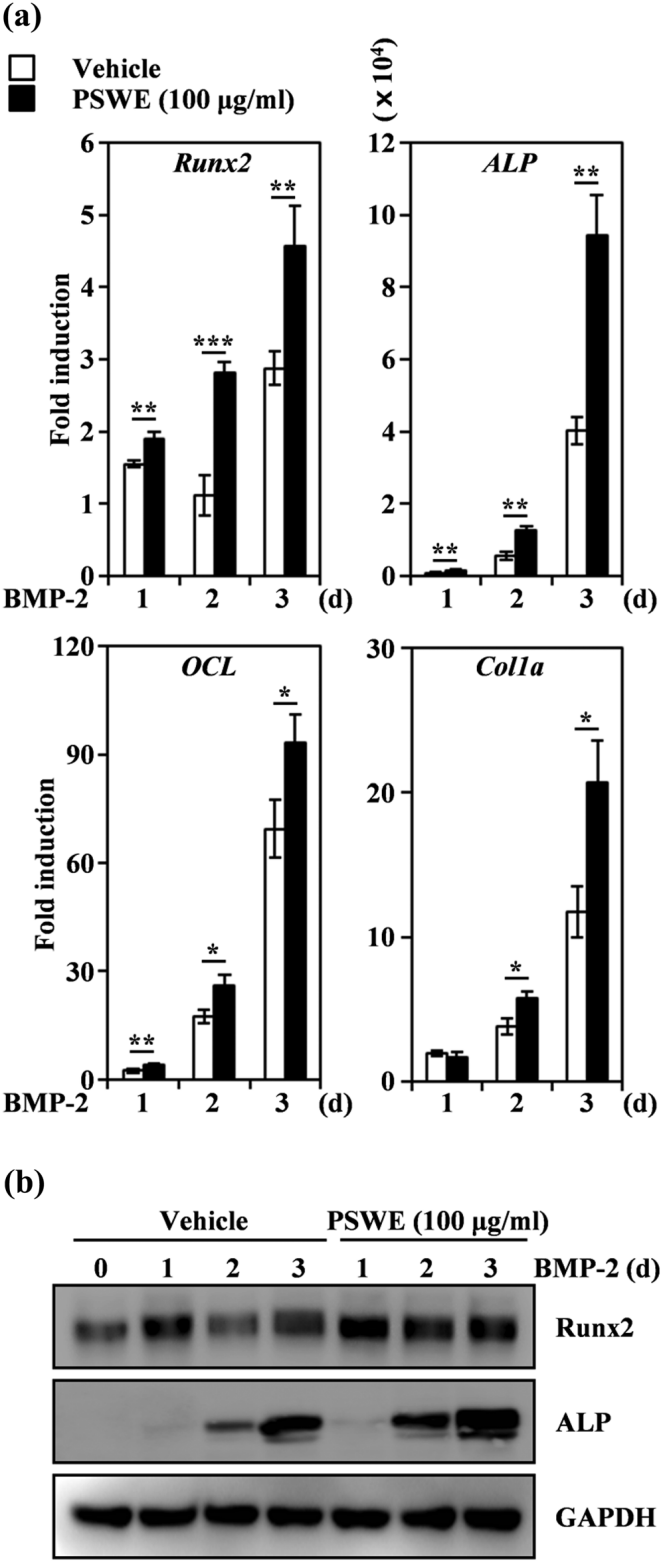
Peanut sprout water extract (PSWE) stimulates bone morphogenetic protein‐2 (BMP‐2)‐induced expression of runt‐related transcription factor 2 (Runx2). (a) C2C12 cells were stimulated in the presence of BMP‐2 (100 ng/ml) with vehicle (water) or PSWE (100 μg/ml) for the indicated times. mRNA expression levels were assessed using real‐time PCR. GAPDH was used as the internal control. * *p* < 0.05; ** *p* < 0.01; *** *p* < 0.001 (versus vehicle control). (b) Effects of PSWE on the levels of Runx2 and alkaline phosphatase were evaluated by immune blot analysis. GAPDH was used as the internal control. One representative result from three independent experiments yielding similar results is shown

Next, we examined the stimulatory effect of PSWE on osteoblast differentiation by evaluating the expression of several osteogenesis‐related genes, including transcription factors. The BMP‐2‐induced levels of mRNAs encoding osteogenesis‐related transcription factors, including Runx2, were synergistically increased by the addition of PSWE, and transcriptional factor‐regulated molecules such as ALP, osteocalcin (OCL), and collagen type I, alpha (Col1a) also significantly stimulated mRNA induction by PSWE at the indicated times (Figure 2a). Western blot analysis confirmed that the BMP‐2‐induced expression of Runx2 and ALP proteins was synergistically enhanced by PSWE treatment (Figure 2b). Together, these results suggest that the osteogenic activity of PSWE arises from its ability to enhance the expression of Runx2, which is required for osteoblast differentiation.

### 3.3 PSWE contributes to BMP‐2‐mediated activation of the AKT/MAP kinase signaling pathways

**Figure 3:**
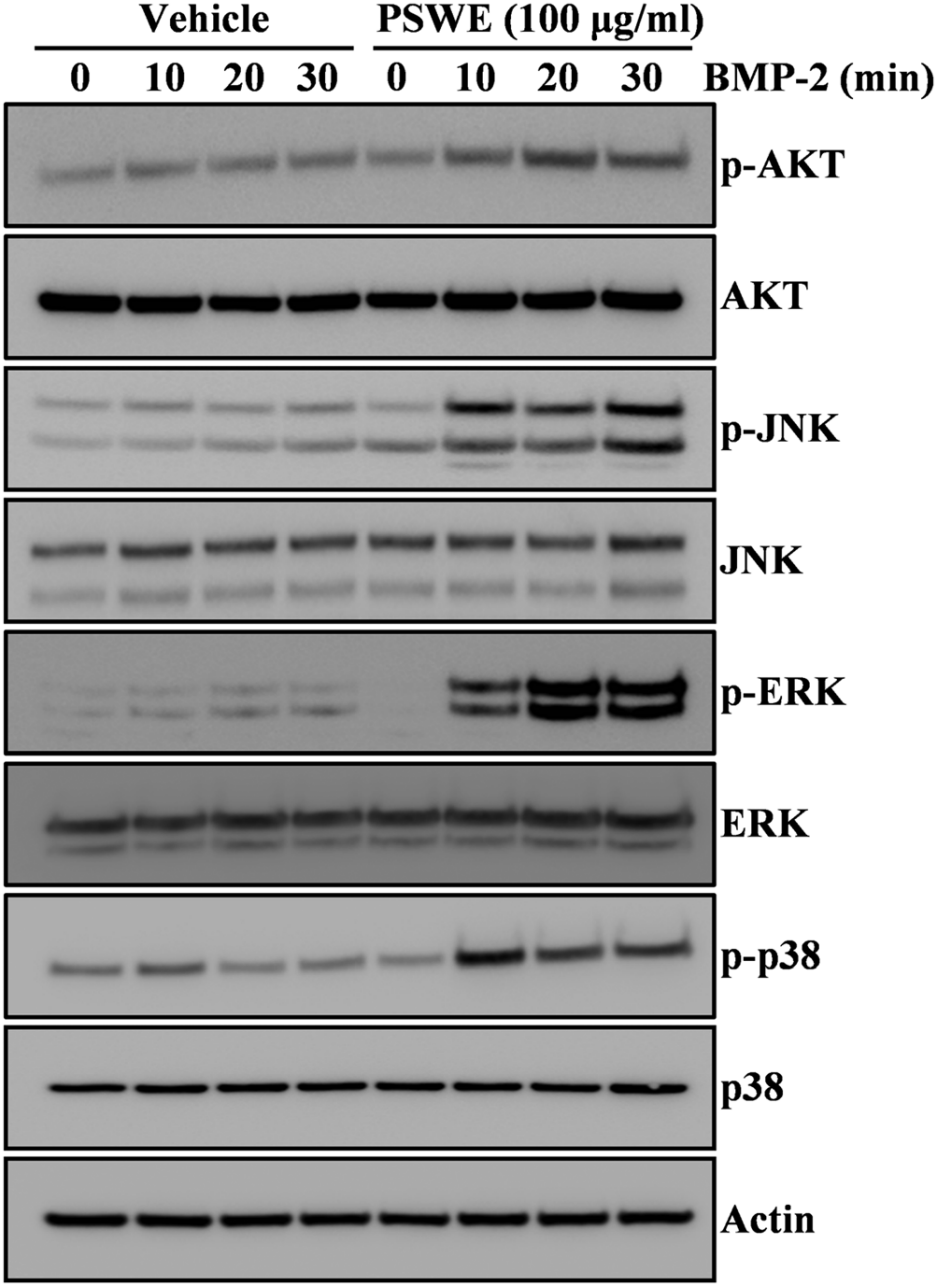
Peanut sprout water extract (PSWE) induces bone morphogenetic protein‐2 (BMP‐2)‐mediated phosphorylation ofAKT/MAP kinase signaling molecules. Following serum starvation for 1 day, C2C12 cells were pretreated with vehicle or PSWE (100 μg/ml) for 1 hr prior to BMP‐2 stimulation (100 ng/ml) for the indicated times. The expression levels of the signaling molecules were evaluated by Western blotting. Actin was used as the internal control

To elucidate the mechanism underlying the anabolic activity of PSWE, we investigated whether PSWE could affect the induction of BMP‐2‐related signaling pathways associated with the regulation of Runx2, a master transcription factor. The addition of PSWE synergistically increased the BMP‐2‐induced phosphorylation of RAC‐Alpha Serine/Threonine‐Protein Kinase (AKT) and MAP kinases, including extracellular signal‐regulated kinase (ERK), c‐Jun N‐terminal kinase (JNK), and p38 (Figure 3), whereas PSWE treatment did not affect the BMP‐2‐induced phosphorylation of Smad (Figure S4).

### 3.4 Identification and characterization of soyasaponin Bb, a major component of PSWE

**Figure 4:**
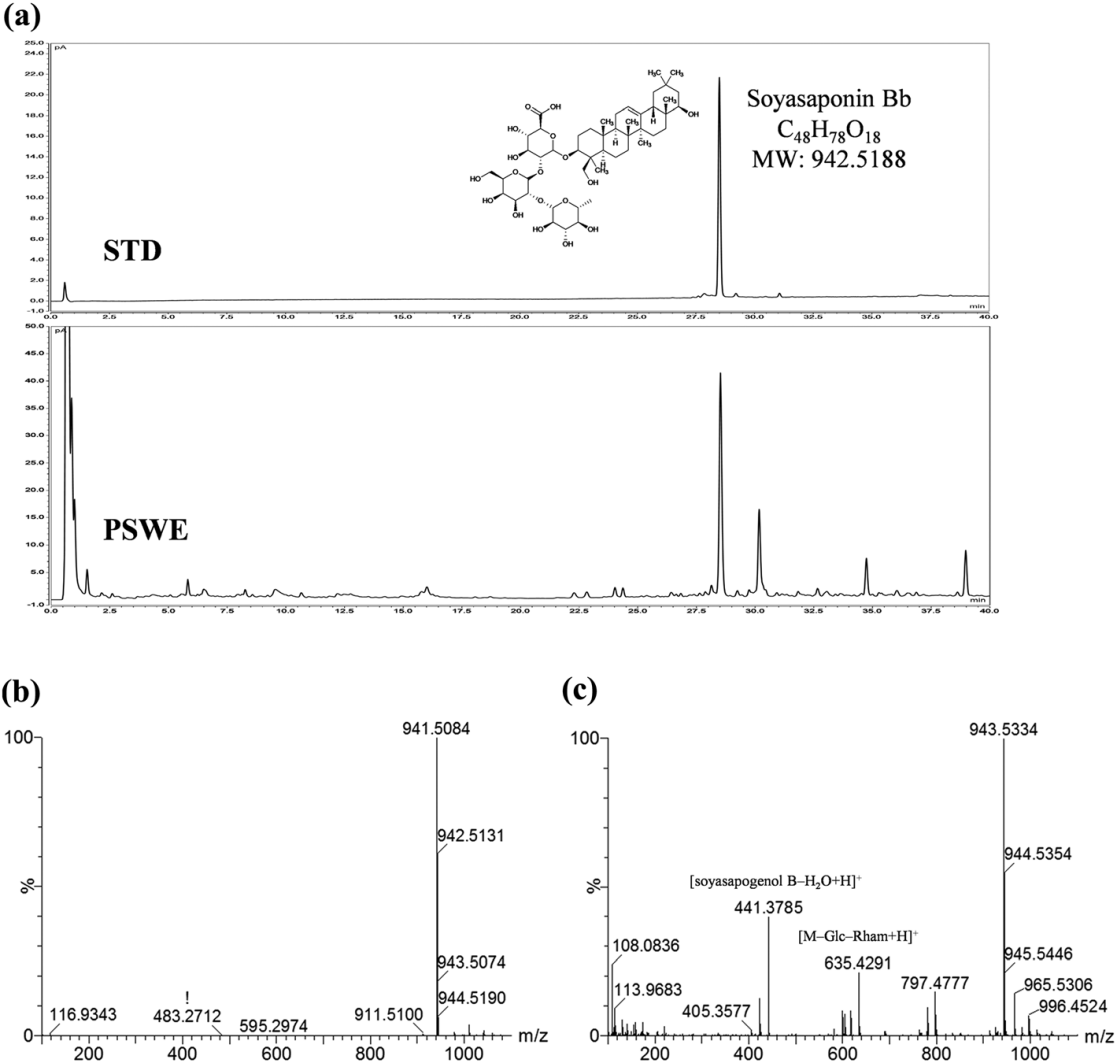
Chemical structure and identification of soyasaponin Bb in peanut sprout water extract (PSWE) by ultra‐high performance liquid chromatography‐charged aerosol detection (UHPLC‐CAD) and UPLC‐Q‐TOF/MS. (a) UHPLC‐CAD chromatogram of soyasaponin Bb STD and PSWE. (b and c) UPLC‐Q‐TOF/MS base peak intensity chromatogram of negative (b) and positive (c) ion modes for identification of soyasaponin Bb

To identify the bioactive compound in PSWE, we investigated its chemical composition by UHPLC‐CAD and UPLC‐Q‐TOF/MS. The structure of soyasaponin Bb was established by comparing its retention times to those of an authentic standard and by comparing the MS/MS spectra and fragmentation pattern with values reported in the literature (Jin, Yang, Su, & Ren, 2007). On the basis of the comparison with the standard, the major peak in PSWE was identified as soyasaponin Bb (Figure 4a). We further confirmed the presence of soyasaponin Bb in PSWE by analyzing UPLC‐Q‐TOF/MS data. In the ESI spectra, soyasaponin Bb generated a high abundance of deprotonated ions [M–H]^−^ at *m/z* 941.5084, corresponding to the formula C_48_H_77_O_18_ (error, −2.8 ppm), in negative mode (Figure 4b), and [M + H]^+^ ions at 943.5334, corresponding to the formula C_48_H_79_O_18_ (error, 7.2 ppm), in positive mode (Figure 4c). As shown in Figure 4c, soyasaponin Bb yielded the product ion [M + H]^+^ at *m/z* 797.4777 via the loss of the terminal rhamnose unit. Another characteristic fragment ion, [M–Glc–Rham+H]^+^ at *m/z* 635.4291 and the aglycone soyasapogenol B [soyasapogenol B–H_2_O + H]^+^ at *m/z* 441.3785, were also observed.

### 3.5 Soyasaponin Bb enhances BMP‐2‐dependent osteogenesis by activating Runx2

**Figure 5:**
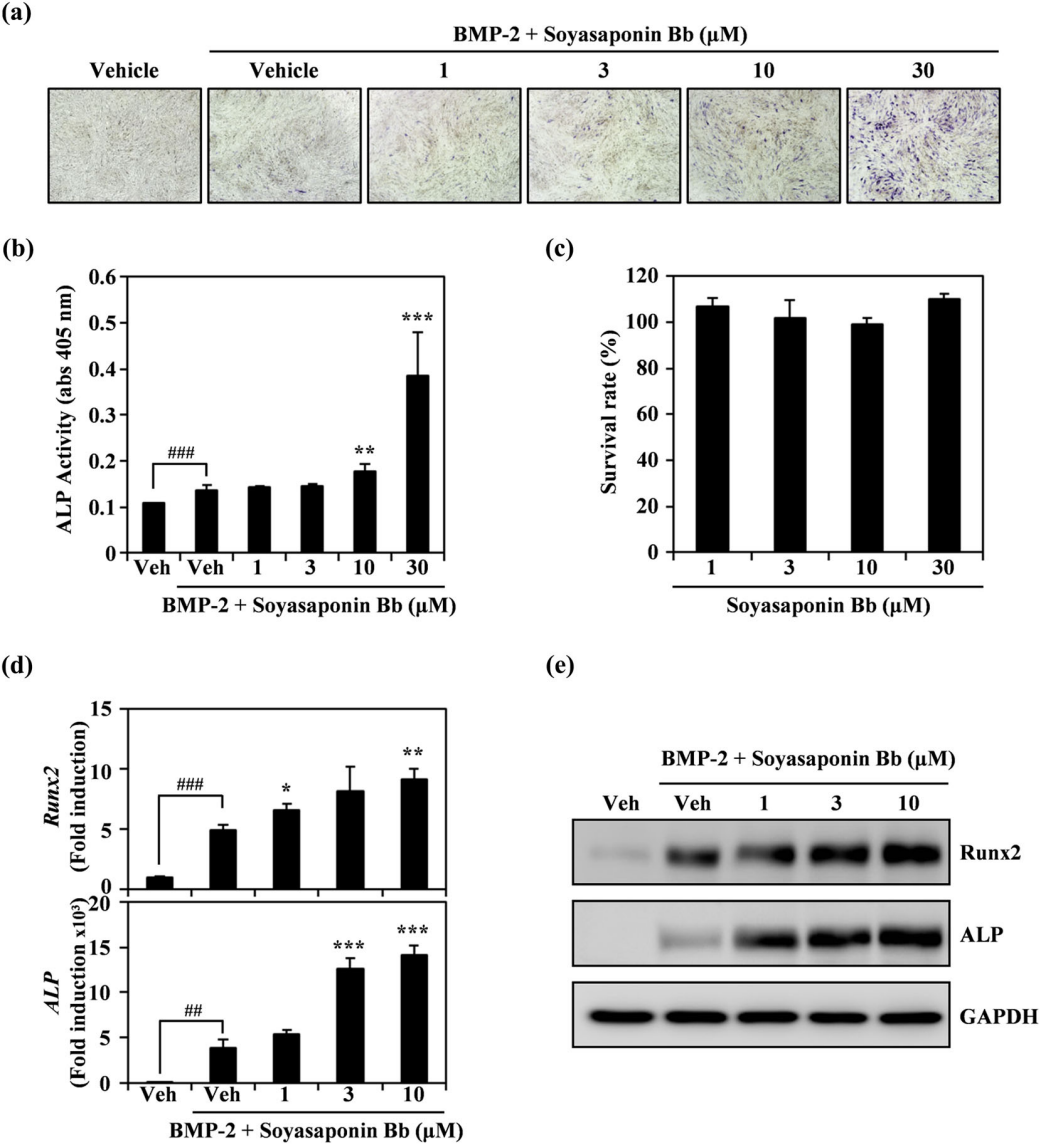
Soyasaponin Bb in peanut sprout water extract stimulates osteogenesis by inducing runt‐related transcription factor 2. (a) Effect of soyasaponin Bb on bone morphogenetic protein‐2 (BMP‐2)‐induced alkaline phosphatase (ALP) staining. Image was acquired under a light microscope (magnification, ×100). (b) ALP activity was measured. ^###^ *p* < 0.001 (versus control); ** *p* < 0.01; *** *p* < 0.001 (versus BMP‐2‐treated group). (c) Cell viability was determined using the CCK‐8 assay. (d, e) The indicated mRNA and protein expression levels were evaluated at the indicated concentrations by real‐time PCR or immunoblotting. Data are expressed as fold change in mRNA level relative to control. ^##^ *p* < 0.01; ^###^ *p* < 0.001 (versus control); * *p* < 0.05; ** *p* < 0.01; *** *p* < 0.001 (versus BMP‐2‐treated group). Data are representative of at least three experiments [Colour figure can be viewed at wileyonlinelibrary.com]

To determine whether identified soyasaponin Bb from PSWE is the component responsible for improving osteoblast differentiation, we evaluated its effect on BMP‐2‐mediated commitment of C2C12 cells into osteoblasts by monitoring the expression and activity of ALP and the expression of transcription factors involved in osteogenesis. In a dose‐dependent manner, soyasaponin Bb significantly enhanced the BMP‐2‐induced expression of ALP expression (Figure 5a). In addition, soyasaponin Bb enhanced osteoblast differentiation and mineralization in MC3T3‐E1 and C2C12 cells (Figure S5). Moreover, soyasaponin Bb also increased the activity of ALP, a biomarker of osteoblast differentiation, in the presence of BMP‐2 (Figure 5b). However, soyasaponin Bb did not affect the survival of C2C12 cells, indicating that the anabolic action of soyasaponin Bb was not due to cytotoxic effects (Figure 5c). To further evaluate the osteogenic action of soyasaponin Bb, we monitored the expression of osteogenic molecules by real‐time PCR and immunoblotting. As shown in Figure 5d, treatment with soyasaponin Bb dramatically induced the mRNA expression of Runx2 and ALP on Day 3 of differentiation. In addition, addition of soyasaponin Bb for 3 days significantly and dose‐dependently enhanced the expression of Runx2 and ALP proteins in the presence of BMP‐2 (Figure 5e). Taken together, these results demonstrate that soyasaponin Bb isolated from PSWE is the bioactive ingredient responsible for acceleration of osteoblast differentiation.

### 3.6 Soyasaponin Bb content of PSWE and its anabolic activity depend on the germination period

**Figure 6:**
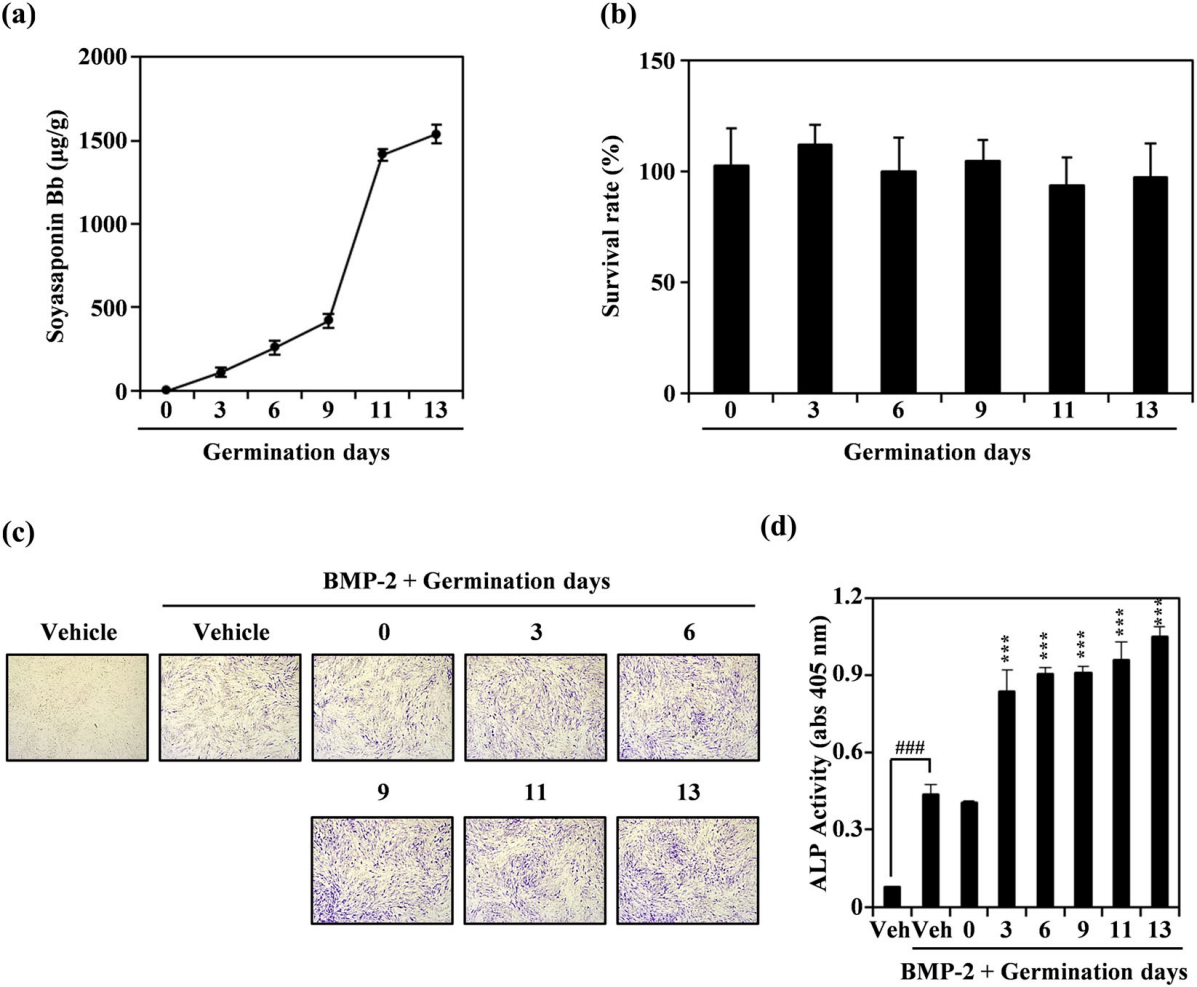
Soyasaponin Bb content and osteogenic activity of peanut sprout water extract (PSWE) increase with germination time. (a) Change in soyasaponin Bb content of PSWE with germination period. (b) Effect of PSWE (30 μg/ml) on C2C12 cell viability. (c, d) The effect of varying the germination period (30 μg/ml) on bone morphogenetic protein‐2 (BMP‐2)‐induced alkaline phosphatase (ALP) staining was visualized and evaluated by measuring ALP activity. ^###^ *p* < 0.001 (versus control); *** *p* < 0.001 (versus BMP‐2‐treated group). Data are expressed as means ± *SD* and are representative of at least three experiments [Colour figure can be viewed at wileyonlinelibrary.com]

To obtain further insight into how soyasaponin Bb enhanced osteoblast differentiation, we investigated soyasaponin Bb content and the associated effect on anabolic activity, over the course of sprouting. As shown in Figure 6a and Figure S6, the content of soyasaponin Bb increased remarkably with sprout growth time. At noncytotoxic concentrations (≤30 μg/ml; Figure 6b), PSWE significantly enhanced BMP‐2‐stimulated osteogenic differentiation in a manner dependent on sprouting time (Figure 6c). In addition, ALP activity was further stimulated by increasing the germination period (Figure 6d). These results suggest that PSWE by increasing sprouting time could improve osteogenic activity by inducing soyasaponin Bb content.

**Figure 7:**
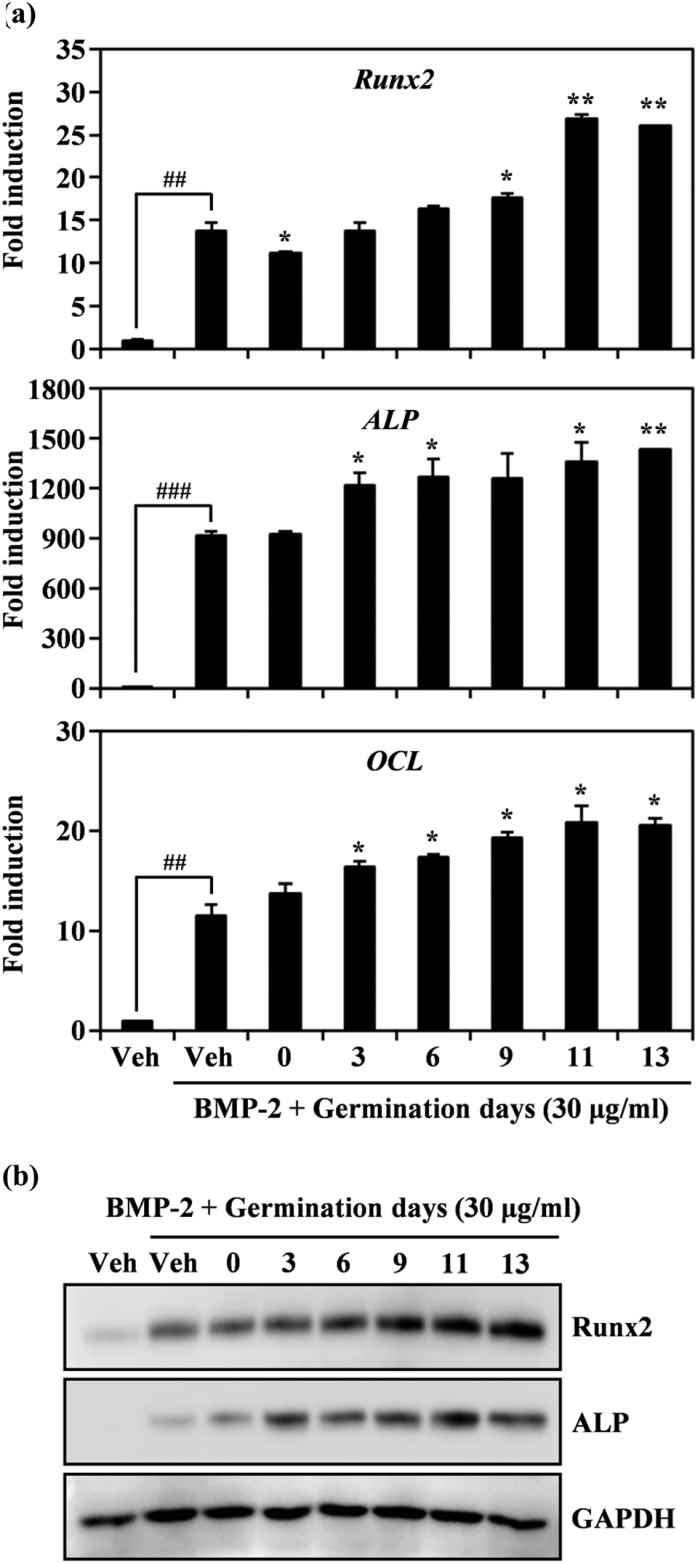
Peanut sprout water extract (PSWE) stimulates runt‐related transcription factor 2 and alkaline phosphatase expression as a function of the length of the germination period. (a) The effect of PSWE on bone morphogenetic protein‐2 (BMP‐2)‐induced mRNA expression was analyzed by real‐time PCR as described in Figure 2a. Glyceraldehyde 3‐phosphate dehydrogenase was used as the internal control. ^##^ *p* < 0.01; ^###^ *p* < 0.001 (versus control); * *p* < 0.05; ** *p* < 0.01 (versus bone morphogenetic protein‐2 [BMP‐2]‐treated group). Data are representative of at least three experiments. (b) Effects of PSWE on BMP‐2‐stimulated protein levels were evaluated using Western blot analysis. One representative result from three independent experiments yielding similar results is shown

To elucidate the relationship between anabolic activity and peanut sprouting time, we examined the expression pattern of several BMP‐2‐dependent molecules on different sprouting days. On differentiation Day 3, the addition of BMP‐2 induced the expression of osteogenic mRNAs including Runx2, ALP, and OCL, and its induction was further amplified by PSWE with increased sprouting times (Figure 7a). Furthermore, osteogenesis‐related protein expression was synergistically induced by increasing the germination time (Figure 7b). These results show that PSWE made from sprouts with a longer sprouting period significantly enhanced BMP‐2‐mediated induction of Runx2, a transcription factor that is required for osteogenic activity.

## 4 DISCUSSION

This study is the first to show that PSWE and soyasaponin Bb PSWE increases BMP‐2‐dependent osteoblast differentiation by inducing ALP expression and activity without apparent cytotoxicity. Previous work has shown that BMP‐2 promotes the commitment of the pluripotent mesenchymal precursor cell line, C2C12, into preosteoblasts capable of bone formation and mineralization. In addition, the BMP‐2‐induced commitment of C2C12 cells into osteoblasts leads to ALP expression, an early marker of osteoblastic differentiation.

Furthermore, we found that PSWE dramatically enhanced mRNA and protein expression of Runx2 during BMP‐2‐dependent osteoblast differentiation, suggesting that PSWE stimulates BMP‐2‐dependent osteoblast differentiation by inducing osteoblast‐specific transcription factors such as Runx2, which is induced during BMP‐2‐stimulated transdifferentiation of C2C12 cells and involved in the development of osteoblastic cells and bone formation (Wang et al., 2006). Consistent with this, PSWE treatment also induced the expression of BMP‐2‐induced Runx2 downstream molecules, such as ALP, OCL, and Col1a, which are known osteoblast‐specific molecules (Li, Felber, Elks, Croucher, & Roehl, 2009; Lu, Robertson, & Brennan, 2004).

The effects of PSWE on osteogenic differentiation enhanced us to investigate BMP‐2‐related signaling pathways. BMP‐2 activates AKT, MAP kinases, and Smad molecules in osteoblastic cells. Interestingly, PSWE treatment did not affect the phosphorylation of Smad, a major BMP‐2‐dependent signaling molecule required for osteogenesis but activated AKT and MAP kinases, which are downstream components of the Ras–PI3K signaling pathway whose activation promotes osteoblast differentiation (Ghosh‐Choudhury, Mandal, & Choudhury, 2007). Furthermore, BMP‐2 stimulation of AKT and MAP kinases leads to activation of Runx2 (Bokui et al., 2008; Mukherjee, Wilson, & Rotwein, 2010). Therefore, these data suggest that PSWE might enhance osteogenic differentiation by activating the AKT/MAP–Runx2 signaling pathway.

Saponins are present in hundreds of different types of plants and foods including beans, chickpeas, peanuts, quinoa, and soy. In the Fabaceae, soyasaponins can be classified into Groups A and B according to their aglycone structure, that is, the presence of Soyasapogenol A or B (Zhang & Popovich, 2009). Interestingly, we found that PSWE with osteogenic activity contained high levels of soyasaponin Bb, which has anti‐oxidative, anti‐carcinogenic, cardiovascular protective, and hepatoprotective effects (Gurfinkel & Rao, 2003; Jiang, Zhong, Qi, & Ma, 1993; Kinjo et al., 2003; Lee, Park, Yeo, Han, & Kim, 2010). However, its osteogenic activity has not been previously reported. In this study, we showed that soyasaponin Bb in PSWE enhanced BMP‐2‐dependent osteogenesis in a dose‐dependent manner via induction of Runx2 and ALP, suggesting that soyasaponin Bb in PSWE could be the bioactive substance responsible for BMP‐2‐induced osteoblast differentiation.

Since sprouting can generate bioactive compounds, thereby increasing the health‐boosting biological effects of seeds (Chavan & Kadam, 1989; Kim, Jeong, Gorinstein, & Chon, 2012), we investigated soyasaponin Bb content, as well as its mode of anabolic action, as a function of sprouting period. The results of this analysis revealed that the concentration of soyasaponin Bb in PSWE increased with sprouting time. Moreover, the osteogenic activity of PSWE, including its effect on the BMP‐2‐mediated expression of Runx2, ALP, and OCL, was dependent on its soyasaponin Bb content. The results suggest that the specific osteogenic action of PSWE can be attributed to the presence and concentration of soyasaponin Bb.

## 5 CONCLUSIONS

To the best of our knowledge, this study is the first to demonstrate that PSWE and its phytochemical soyasaponin Bb have anabolic potential in BMP‐2‐mediated osteoblast differentiation. Specifically, we found that PSWE and soyasaponin Bb were associated with induction of the MAP kinase–Runx2 signaling pathways required for osteoblast differentiation. Induction of Runx2 leads to the expression of factors required for bone formation, including ALP, OCL, and Col1a. In addition, the osteogenic activity of PSE is determined by the content of soyasaponin Bb. Although a detailed in vivo experiment and clinical study of the bone formation activity of PSWE should be carried out before it is applied to humans, the results presented here suggest that PSWE and soyasaponin Bb could be useful in the development of functional food and therapeutic agents for preventing and treating osteoblast‐related bone metabolic disorders.

## CONFLICT OF INTEREST

The authors have declared no conflict of interest.

## Supporting information

Table S1. Primers used in this study
Figure S1. PSWE enhances BMP‐2–induced osteoblast differentiation in MC3T3‐E1 and primary osteoblast cells. MC3T3‐E1 (subclone 4) and pOB (mice calvaria primary osteoblasts) cells were stimulated in the presence of BMP‐2 (100 ng/ml) with vehicle (water) or the indicated concentrations of PSWE. The cells were cultured for 6 days and osteoblast differentiation was visualized by ALP staining.
Figure S2. PSWE stimulates bone mineralization. Osteogenic activities of PSWE were revealed by and Alizarin red staining in the presence of ascorbic acid and β‐glycerophosphate (AA, β‐GP), or BMP‐2.
Figure S3. PSWE was not enhanced osteoblast differentiation in the absence of BMP‐2. MC3T3‐E1 cells were visualized by ALP and Alizarin Red staining (AZ) with the indicated concentrations of PSWE.
Figure S4. PSWE does not affect BMP‐2–induced phosphorylation of Smad. Following serum starvation for 1 day, C2C12 cells were pre‐treated with vehicle or PSWE (100 μg/ml) for 1 hr prior to BMP‐2 simulation (100 ng/ml) for the indicated times. Expression of Smad molecules was evaluated by Western blot analysis. One representative result from three independent experiments yielding similar results is shown.
Figure S5. Soyasaponin Bb enhanced osteoblast differentiation and mineralization in MC3T3‐E1 and C2C12 cells. (A) MC3T3‐E1 cells were cultured for 6 days with the indicated concentrations of soyasaponin Bb in the presence of BMP‐2 or AA + β‐GP. After cell fixation, ALP expression was visualized by ALP staining. (B) Effect of soyasaponin Bb on mineralization was evaluated by staining with 2% Alizarin Red solution.
Figure S6. Comparison of UPLC chromatograms of soyasaponin Bb in PSWE from sprouts harvested at different times: seeds/0 days (A); 3 days (B); 6 days (C); 9 days (D); 11 days (E); and 13 days (F).
