# Supplementary material for: Biofunctional soyasaponin Bb in peanut (Arachis hypogaea L.) sprouts enhances bone morphogenetic protein‐2‐dependent osteogenic differentiation via activation of runt‐related transcription factor 2 in C2C12 cells
Source: Phytother Res. 2019 Mar 18;33(5):1490–500. doi: 10.1002/ptr.6341 (PMC6593731; doi:10.1002/ptr.6341)
Supplement: Supplementary file 1 — Table S1. Primers used in this study Figure S1. PSWE enhances BMP‐2–induced osteoblast differentiation in MC3T3‐E1 and primary osteoblast cells. MC3T3‐E1 (subclone 4) and pOB (mice calvaria primary osteoblasts) cells were stimulated in the presence of BMP‐2 (100 ng/ml) with vehicle (water) or the indicated concentrations of PSWE. The cells were cultured for 6 days and osteoblast differentiation was visualized by ALP staining. Figure S2. PSWE stimulates bone mineralization. Osteogenic activities of PSWE were revealed by and Alizarin red staining in the presence of ascorbic acid and β‐glycerophosphate (AA, β‐GP), or BMP‐2. Figure S3. PSWE was not enhanced osteoblast differentiation in the absence of BMP‐2. MC3T3‐E1 cells were visualized by ALP and Alizarin Red staining (AZ) with the indicated concentrations of PSWE. Figure S4. PSWE does not affect BMP‐2–induced phosphorylation of Smad. Following serum starvation for 1 day, C2C12 cells were pre‐treated with vehicle or PSWE (100 μg/ml) for 1 hr prior to BMP‐2 simulation (100 ng/ml) for the indicated times. Expression of Smad molecules was evaluated by Western blot analysis. One representative result from three independent experiments yielding similar results is shown. Figure S5. Soyasaponin Bb enhanced osteoblast differentiation and mineralization in MC3T3‐E1 and C2C12 cells. (A) MC3T3‐E1 cells were cultured for 6 days with the indicated concentrations of soyasaponin Bb in the presence of BMP‐2 or AA + β‐GP. After cell fixation, ALP expression was visualized by ALP staining. (B) Effect of soyasaponin Bb on mineralization was evaluated by staining with 2% Alizarin Red solution. Figure S6. Comparison of UPLC chromatograms of soyasaponin Bb in PSWE from sprouts harvested at different times: seeds/0 days (A); 3 days (B); 6 days (C); 9 days (D); 11 days (E); and 13 days (F). [file PTR-33-1490-s001.docx]

**Supplementary Information**

**Bio-functional soyasaponin Bb in peanut (*Arachis hypogaea L.*) sprouts enhances BMP-2–dependent osteogenic differentiation via activation of Runx2 in C2C12 cells**

Shin-Hye Kim^a,b,1^, Heung Joo Yuk^c,1^, Hyung Won Ryu^d^, Sei-Ryang Oh^d^, Duk Young Song^e^, Kwang-Sik Lee^e,f^, Kie-In Park^b^, Sik-Won Choi^a,^*, Woo Duck Seo^e,^*

^a^Forest Biomaterials Research Center, National Institute of Forest Science (NIFS), Jinju, Gyeongnam 52817, Korea; ^b^Department of Biological Sciences, College of Natural Science, Chonbuk National University, Jeonju 54896, Korea; ^c^Korean Medicine Convergence Research Division, Korea Institute of Oriental Medicine (KIOM), Daejeon 34054, Korea; ^d^Natural Medicine Research Center, Korea Research Institute of Bioscience & Biotechnology, Cheongju 28116, Korea; ^e^Division of Crop Foundation, National Institute of Crop Science (NICS), Rural Development Administration (RDA), Wanju 55365, Korea; ^f^College of Crop Science and Biotechnology, Dankook University, Cheonan 31116, Korea

^1^Shin-Hye Kim and Heung Joo Yuk contributed equally to this work.

***Co-corresponding author:**

Sik-Won Choi, Ph.D.

Forest Biomaterials Research Center, National Institute of Forest Science (NIFS), Jinju, Gyeongnam 52817, Republic of Korea

Tel: +82-55-760-5093;

Fax: +82-55-759-8432;

E-mail: superwon@korea.kr

Woo Duck Seo, Ph.D.

Laboratory of Crop Resource Development, Division of Crop Foundation, National Institute of Crop Science, Rural Development Administration, Wanju 55365, Republic of Korea

Tel: +82-63-238-5333;

Fax: +82-63-238-5305;

E-mail: swd2002@korea.kr

**Supplementary Table 1. Primers used in this study**

| Target gene | Forward primer (5′🡪3′) | Reverse primer (5′🡪3′) |
| --- | --- | --- |
| *Runx2* | GACTGTGGTTACCGTCATGGC | ACTTGGTTTTTCATAACAGCGGA |
| *ALP* | GATGGCGTATGCCTCCTGCA | CGGTGGTGGGCCACAAAAGG |
| *OCL* | AGGGAAACCTCATCCGTTTG | GAGCCGGAAATAAGGCACAG |
| *Col1a* | CACGAGTCACACCGGAACTT | AGTTTGGGTTGTTCGTCTGT |
| *GAPDH* | ACCACAGTCCATGCCATCAC | TCCACCACCCTGTTGCTGTA |


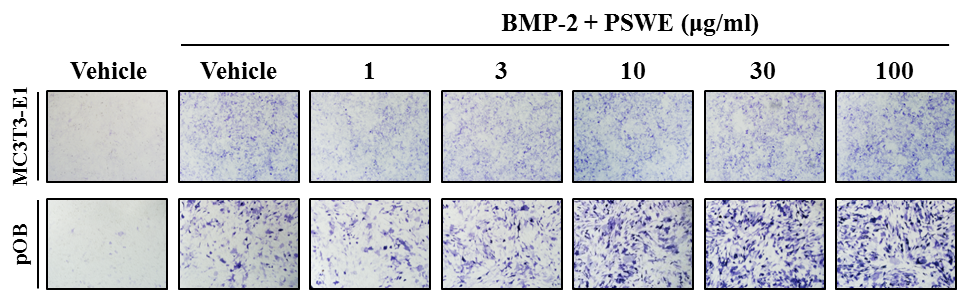


**Supplementary Figure 1. PSWE enhances BMP-2–induced osteoblast differentiation in MC3T3-E1 and primary osteoblast cells.** MC3T3-E1 (subclone 4) and pOB (mice calvaria primary osteoblasts) cells were stimulated in the presence of BMP-2 (100 ng/ml) with vehicle (water) or the indicated concentrations of PSWE. The cells were cultured for 6 days and osteoblast differentiation was visualized by ALP staining.

**
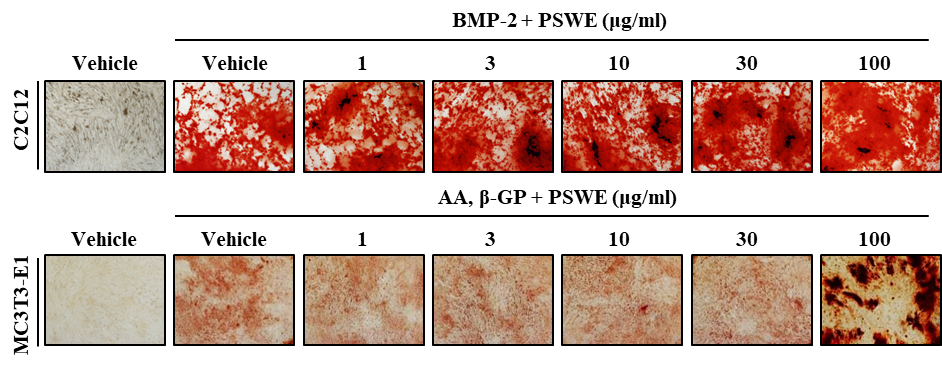
**

**Supplementary Figure 2. PSWE stimulates bone mineralization.** Osteogenic activities of PSWE were revealed by and Alizarin red staining in the presence of ascorbic acid and β-glycerophosphate (AA, β-GP), or BMP-2.


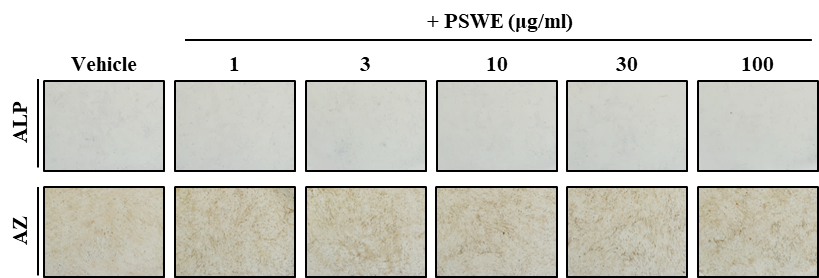


**Supplementary Figure 3. PSWE was not enhanced osteoblast differentiation in the absence of BMP-2.** MC3T3-E1 cells were visualized by ALP and Alizarin Red staining (AZ) with the indicated concentrations of PSWE.

**
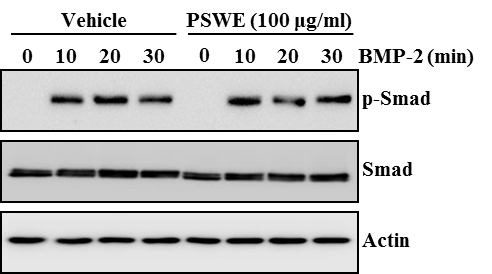
**

**Supplementary Figure 4. PSWE does not affect BMP-2–induced phosphorylation of Smad.** Following serum starvation for 1 day, C2C12 cells were pre-treated with vehicle or PSWE (100 μg/ml) for 1 hr prior to BMP-2 simulation (100 ng/ml) for the indicated times. Expression of Smad molecules was evaluated by Western blot analysis. One representative result from three independent experiments yielding similar results is shown.

**
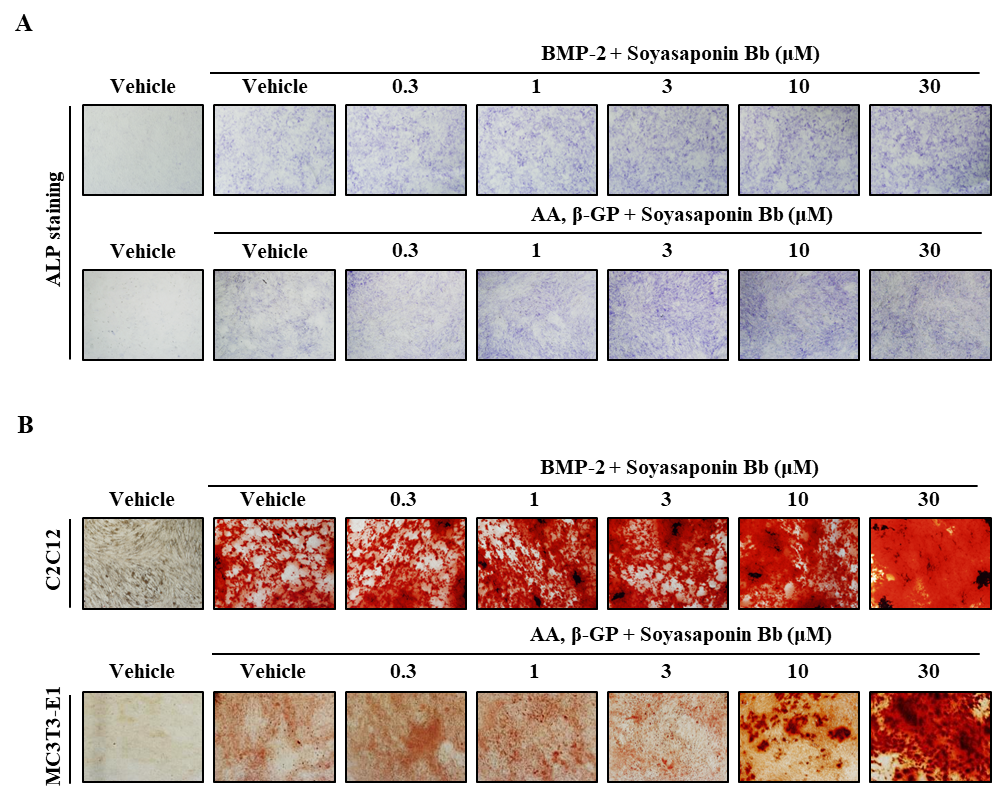
**

**Supplementary Figure 5. Soyasaponin Bb enhanced osteoblast differentiation and mineralization in MC3T3-E1 and C2C12 cells.** (A) MC3T3-E1 cells were cultured for 6 days with the indicated concentrations of soyasaponin Bb in the presence of BMP-2 or AA + β-GP. After cell fixation, ALP expression was visualized by ALP staining. (B) Effect of soyasaponin Bb on mineralization was evaluated by staining with 2% Alizarin Red solution.


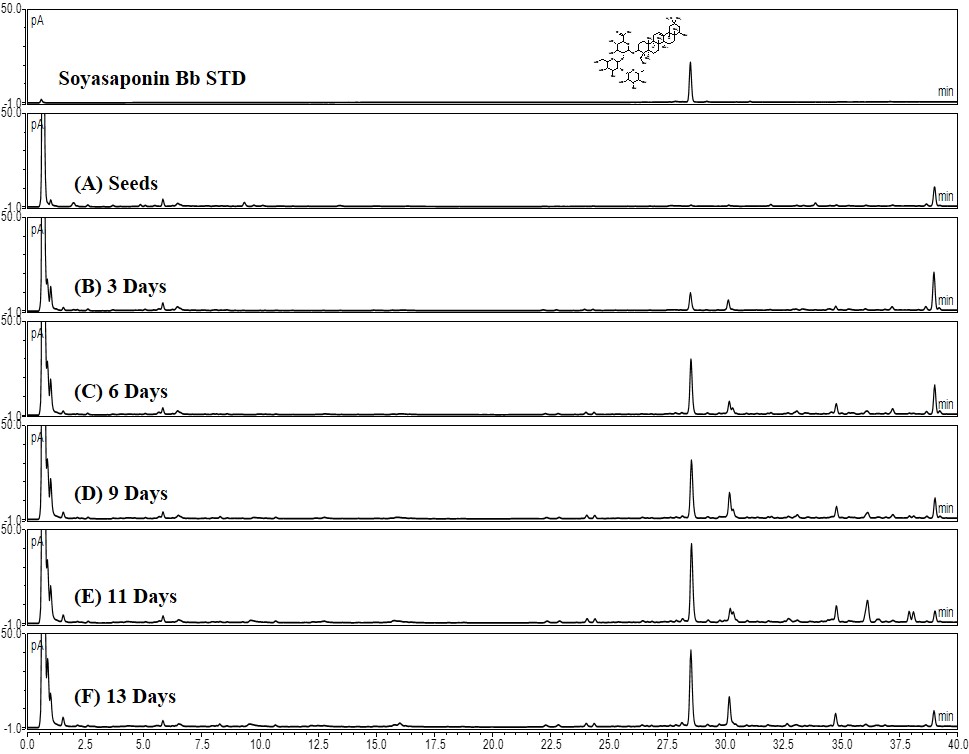


**Supplementary Figure 6.** Comparison of UPLC chromatograms of soyasaponin Bb in PSWE from sprouts harvested at different times: seeds/0 days (A); 3 days (B); 6 days (C); 9 days (D); 11 days (E); and 13 days (F).
